# Whole blood assay as a model for *in vitro* evaluation of inflammasome activation and subsequent caspase-mediated interleukin-1 beta release

**DOI:** 10.1371/journal.pone.0214999

**Published:** 2019-04-08

**Authors:** Thi Anh Thu Tran, Hendrika W. Grievink, Katarzyna Lipinska, Cornelis Kluft, Jacobus Burggraaf, Matthijs Moerland, Dimitar Tasev, Karen E. Malone

**Affiliations:** 1 Good Biomarker Sciences, Leiden, the Netherlands; 2 Centre for Human Drug Research, Leiden, the Netherlands; University of the Pacific, UNITED STATES

## Abstract

Processing of pro-interleukin (IL)-1β and IL-18 is regulated by multiprotein complexes, known as inflammasomes. Inflammasome activation results in generation of bioactive IL-1β and IL-18, which can exert potent pro-inflammatory effects. Our aim was to develop a whole blood-based assay to study the inflammasome *in vitro* and that also can be used as an assay in clinical studies. We show whole blood is a suitable milieu to study inflammasome activation in primary human monocytes. We demonstrated that unprocessed human blood cells can be stimulated to activate the inflammasome by the addition of adenosine 5’-triphosphate (ATP) within a narrow timeframe following lipopolysaccharide (LPS) priming. Stimulation with LPS resulted in IL-1β release; however, addition of ATP is necessary for “full-blown” inflammasome stimulation resulting in high IL-1β and IL-18 release. Intracellular cytokine staining demonstrated monocytes are the major producers of IL-1β in human whole blood cultures, and this was associated with activation of caspase-1/4/5, as detected by a fluorescently labelled caspase-1/4/5 probe. By applying caspase inhibitors, we show that both the canonical inflammasome pathway (via caspase-1) as well as the non-canonical inflammasome pathway (via caspases-4 and 5) can be studied using this whole blood-based model.

## Introduction

Inflammasomes are large multimolecular complexes controlling the activation of caspase-1 [1], in response to bacterial and damage-associated stimuli [2, 3]. One of the most studied inflammasome members is nucleotide binding and oligomerization domain and leucine rich-repeat-containing pyrin domain containing 3 (NLRP3) [4]. The NLRP3 complex formation by sequential triggers is known as a canonical pathway of inflammasome activation. The first stimulus initiates nuclear factor-κB (NF-κB) activity by Toll-like receptor (TLR) signaling, thereby inducing *IL1B* and *IL18* mRNA synthesis [5] and licensing the expression of the NLRP3 inflammasome [6]. The second stimulus (for example ATP, potassium efflux, release of mitochondrial DNA, lysosomal damage) leads to the oligomerization and formation of the NLRP3 protein complex, resulting in caspase-1 activation and consequently release of mature IL-1β and IL-18 [7–9]. A so-called ‘non-canonical’ pathway has been identified [10], comprising caspase-11 in mice and caspase-4/5 in humans [11–13], which may produce IL-1β but not IL-18 after a single stimulus. The inflammasome activation is also regulated by caspase-8 that participate as a modulator of canonical NLRP3 signaling [14] or as a trigger of non-canonical IL-1β processing [15].

Mature IL-1β is involved in upregulation of adhesion molecules, induction of chemokines and infiltration of immune cells into tissues [1]. IL-1β–induced pro-inflammatory responses activate host defense during infection while IL-18 drives interferon-gamma (IFNγ) expression in T cells and NK cells [16]. Upregulation of IL-1β expression is observed in multiple disorders including Alzheimer’s disease, diabetes mellitus, atherosclerosis and hypertension [17–19] whereas elevated levels of circulating IL-18 have been reported for patients with heart disease [16] and a role for IL-18 has been suggested in various autoimmune diseases [16, 20, 21]. Given the effector functions of IL-1β and IL-18 and their reported enhanced expression in various pathological conditions, the inhibition of NLRP3 activation or its products may offer a valuable therapeutic approach. The early clinical testing of such agents is hindered by the fact that although IL-18 is constitutively present in plasma [22], NLRP3 activity and IL-1β levels are only increased after stimulation, so assessment of inflammasome inhibition in healthy volunteers is not straightforward. However, NLRP3 inflammasome activity can be induced *ex vivo* in primary human cells, and such models can be employed as pharmacodynamic readout in (pre)clinical pharmacology studies. We previously described the application of caspase-1 inhibitors for inflammasome assays in human whole blood [23]. Other research groups commonly use peripheral blood mononuclear cell (PBMC)-based models or differentiated macrophages when investigating (modulation of) inflammasome activity [24–27]. The secretion of pro-inflammatory cytokines in whole blood in conditions where inflammasome activation is expected has been investigated in the past [28–30] and large differences in variables such as assay matrices, inflammasome triggers, readouts and incubation durations have been reported.

In this study, we thoroughly evaluated the kinetics of inflammasome activation in whole blood, we explored the dynamics of inflammasome stimulation by parallel cytokines, we investigated the potential occurrence of cell death or factors inhibiting inflammasome activity, and we identified by flow cytometry the cell types that are accountable for IL-1β production. We validated our whole blood-based model by application of specific caspase inhibitors, differentiating between the canonical pathway (caspase-1) and the non-canonical pathway (caspase-4/5).

## Materials and methods

### Blood collection

Venous blood was collected from healthy volunteers into sodium-heparin tubes (Becton Dickinson, San Jose, CA, USA) after obtaining written informed consent in accordance with relevant guidelines and regulations. The protocol was approved by the Medical Ethical committee of Leiden University Medical Center.

### *Ex vivo* whole blood culture & stimulation

Cell cultures were performed in an endotoxin-free manner. The blood was stimulated 1:1 volume with either vehicle (CTRL), 2 ng/ml LPS (*Escherichia coli* serotype 0111:B4) or in combination of LPS with ATP (5mM) in duplicate. LPS and ATP were both obtained from Sigma-Aldrich (St Louis, MO, USA). All stimulants were diluted in RPMI 1640 (Gibco, Life technologies) and samples were incubated at 37°C, 5% CO_2_ for 4, 8, 12 and 24h with LPS or LPS+ATP where the ATP was added for the last 30 min of the incubation time. Baseline sample (CTRL 0h) without stimulation 1:1 volume in RPMI 1640 was also processed. NLRP3 inhibition and caspase inhibition was performed with 1h pre-incubation with MCC950 (5 μM), Ac-LEVD-cho and Ac-YVAD-cmk for the indicated concentrations prior to inflammasome stimulation with LPS for 3.5h and ATP for an additional 30 min more. MCC950 is a potent and selective inhibitor of NLRP3 (Invivogen), Ac-LEVD-cho inhibits caspase-4 and 5 (Enzo Lifesciences) and Ac-YVAD-cmk targets caspase 1, 4 and 5 (Sigma-Aldrich). Following incubation, the supernatants of whole blood cultures were collected after centrifugation 2000g for 20 min. Obtained supernatants were frozen on dry ice for 15 min and stored at -80°C for later cytokine analysis. The cells were further processed for flow cytometry analysis.

### Pro-inflammatory cytokines analysis

IL-1β levels from blood culture supernatants were determined by the Meso Scale Discovery platform (human proinflammatory V-Plex plus Kit, MSD,) or Quantikine ELISA (R&D Systems) as indicated in the figure legends. The supernatant levels of tumor necrosis factor alpha (TNFα), IL-6 and IL-8 were determined with the Human Proinflammatory 4-Plex II Ultra-sensitive Kit (Meso Scale Discovery, MSD). IL-18 was measured by ELISA (MBL) or MSD, as indicated in figure legend. IL-18BPa and IL-1sRII levels were determined by ELISA (R&D Systems). ASC/PYCARD was measured by ELISA (Aviva Systems Biology). All measurements were performed according to manufacturer’s instructions.

### Flow cytometry

Following stimulation, 100 μl whole blood sample of each condition (CTRL, LPS, LPS+ATP) was used for flow cytometry. Red blood cells were lysed using 2 ml RBC lysis buffer (Biolegend), incubated at room temperature for 10 min, followed by centrifugation at 350g for 5 min. The samples were washed twice in FACS buffer (PBS + 0.5% BSA) and stained with 5 μl of the following antibodies anti-CD3-FITC (clone OKT3), anti-CD14-FITC (clone M5E2), anti-CD20-FITC (clone 1412), anti-CD15-PE/Cy7 (clone W6D3), anti-CD16-APC/Cy7 (clone 3G8), anti-CD45-pacific blue (clone HI30) (all from Biolegend), anti-CD20-APC (clone 2H7, eBioscience), 1 μl antiCD56-APC (clone CMSSB, eBioscience), 1 μl anti-HLA-DR-PerCy5.5 (clone G46-6, Becton Dickinson), CD123-PE (clone 9F5, BD) and 1 μl CD11c-FITC (clone B-ly6, BD) by incubation for 30 min at 4°C in the dark. To check for cell viability an APC Annexin V Apoptosis Detection Kit with PI (Biolegend,) was used following manufacturer’s instructions. Intracellular IL-1β and ASC staining were done in the presence of a protein transport inhibitor cocktail containing both brefeldin A and monensin (eBioscience). The samples were fixed and permeabilized with fixation buffer (BioLegend) and intracellular permeabilization wash buffer (Biolegend), respectively. Blocking was done with Human TruStain FcX (Fc Receptor Blocking Solution) (Biolegend) and then samples were stained with 5 μl IL-1β-PE antibody (clone CRM56, eBioscience) and 1 μl ASC (TMS1)-AF647 (MBL International) for 30 min at 4°C in the dark. Matched isotype controls were used from Biolegend, eBioscience and MBL International. FLICA, FAM-YVAD-FMK, (Immunochemistry technologies) staining was done after red blood cell lysis and surface staining for CD14 was done with anti-CD14-APC (clone M5E2, Becton Dickinson) according to manufacturer’s instructions. The cells were analyzed on a MACSQuant Analyzer 10 flow cytometer (Miltenyi Biotec). Data was analyzed using FlowJo software version 10 (FlowJo LLC). Representative dot plots of gating strategy for assessment of IL-1β expression in monocytes and neutrophils (**S1 Fig panel A**), for monocyte phenotype determination (**S1 Fig panel B**) and for evaluation of IL-1β^+^ cells among monocytes and dendritic cells (**S1 Fig panel C**).

### Statistical analysis

Data are presented as mean value ± standard error of the mean (SEM). Differences in IL-1β release and expression were analyzed by two-way ANOVA and mixed effects model using Tukey’s test for multiple comparisons, respectively. Differences in monocyte marker expression at different time-points were analyzed by mixed effects analysis with Dunnett’s multiple comparison test. Differences was considered significant if p<0.05. For statistical analysis GraphPad Prism version 8.00 software for Windows (GraphPad Software, La Jolla, CA, USA) was used.

## Results

### IL-1β and IL-18 release in whole blood

**Fig 1:**
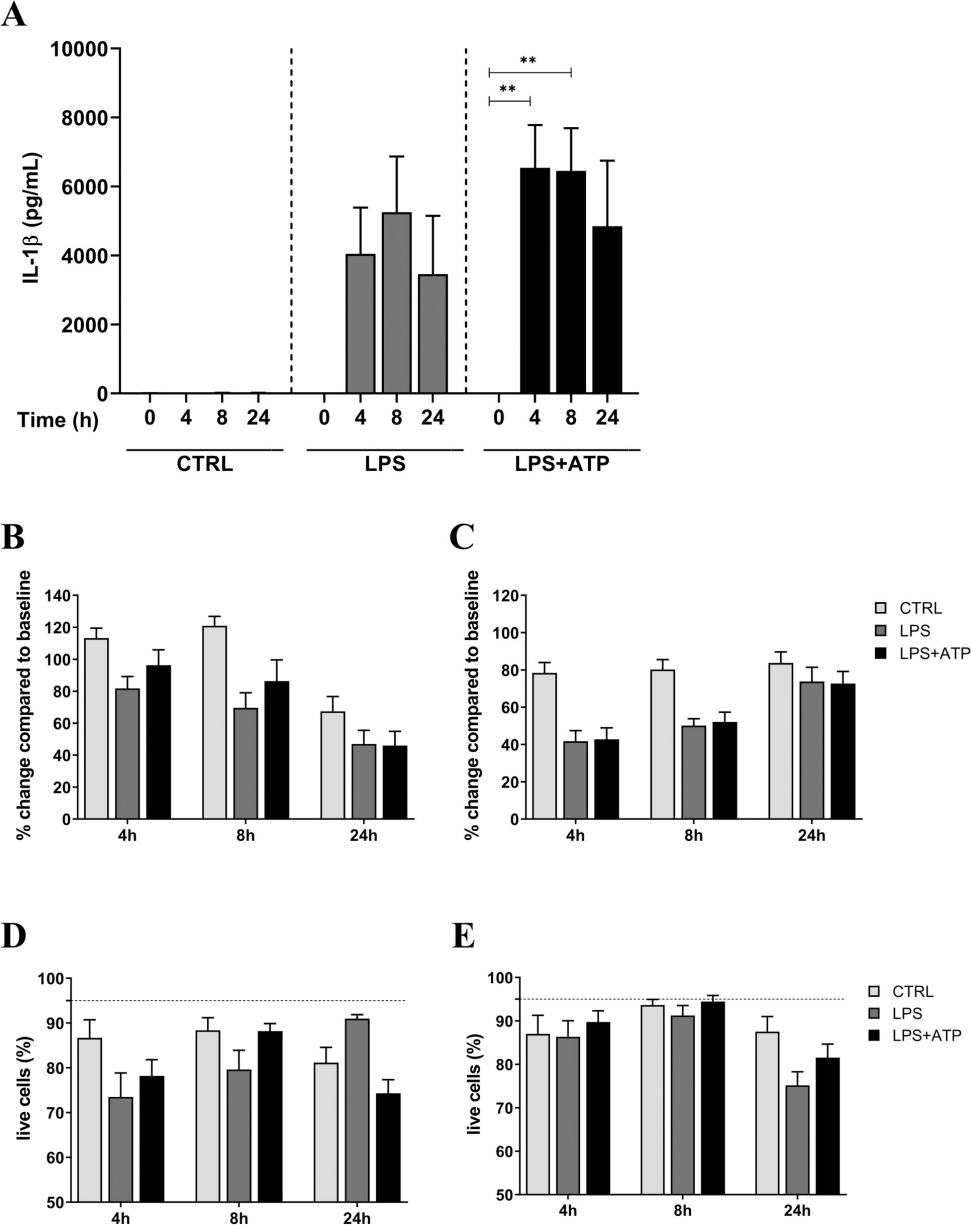
Inflammasome activation in whole blood cultures. (A) IL-1β dynamics and kinetics after inflammasome activation with LPS or LPS+ATP in six donors. **p<0.008 analyzed by two-way ANOVA with Tukey’s multiple comparison test. (B, C) Cellular viability upon inflammasome activation in whole blood cultures. Cell percentage change over the course of 24h stimulation period compared to baseline (CTRL 0h) of monocytes (B) or neutrophils (C). (D, E) Cell viability stained with Annexin V/PI showing percentage of live cells for monocytes (D) and neutrophils (E). no significant differences between CTRL and LPS or LPS+ATP analyzed by mixed effects analysis with Tukey’s multiple comparison test. Data are expressed as mean value ± standard error of the mean (SEM) of each stimulation condition, n = 6; striped line represents baseline values at CTRL 0h.

To compare NLRP3 inflammasome activation of minimally processed whole blood, samples were incubated for 4, 8, 12 and 24h, with LPS (full incubation duration), or LPS (full incubation duration) plus ATP (LPS+ATP; final 30 min of the incubation time). After 4h of incubation with LPS an increase in IL-1β was seen, which was accentuated upon addition of ATP for the final 30 min of the incubation period in six donors (**Fig 1A**). Similar pattern of production was observed for IL-18 in one donor (**S2 Fig**). Substantial levels of TNFα and IL-6 was detected upon the addition of ATP following LPS priming at 12h, while IL-8 did not result in substantial changes to secretion with the addition of ATP. Elevated levels of TNF-a and IL-6 were observed at 12h incubation, thus it is unlikely that these two cytokines are involved in release of IL-1β and IL-18 in the supernatant (**S2 Fig**). After 24h of incubation the release of IL-1β and IL-18 was generally lower after addition of ATP compared to earlier time points (**Fig 1A)**.

The fact that ATP-induced IL-1β and IL-18 release peaked after 4h of LPS priming and decreased upon extended priming suggests that either a loss of specific cell populations occur, or some inhibiting factors may be rapidly up-regulated to counter the responses induced by LPS+ATP. To explore if the loss of specific cell populations might explain the decrease of IL-1β and IL-18 secretion, cell viability was assessed by measuring the number of monocytes and neutrophils in cultures as well as by annexin V/PI staining. The number of viable monocytes in whole blood cultures was slightly decreased at 4 and 8h for stimulated samples while it decreased at the end of incubation period for control as well as stimulated samples (**Fig 1B**). At 4 and 8h a decrease of the neutrophil population is observed for the stimulated conditions but not the control, while this population is restored at 24h (**Fig 1C**). Even though both monocytes and neutrophils cell numbers decreased during stimulation no statistically significant differences were observed. Furthermore, the staining with annexin V showed that still 75–100% of the monocytes and neutrophils were viable during the 24h experimental procedures (**Fig 1D and 1E**).

To test the second hypothesis whether inhibiting factors may dampen the whole blood responses induced by LPS+ATP, soluble IL-1 receptor II (IL-1sRII) and IL-18 binding protein a (IL-18BPa) levels were measured in supernatants of whole blood cultures, and none of these negative regulators exhibited changes coinciding with observed IL-1β and IL-18 responses, (**S3 Fig**).

### Monocytes are a major source of IL-1β

**Fig 2:**
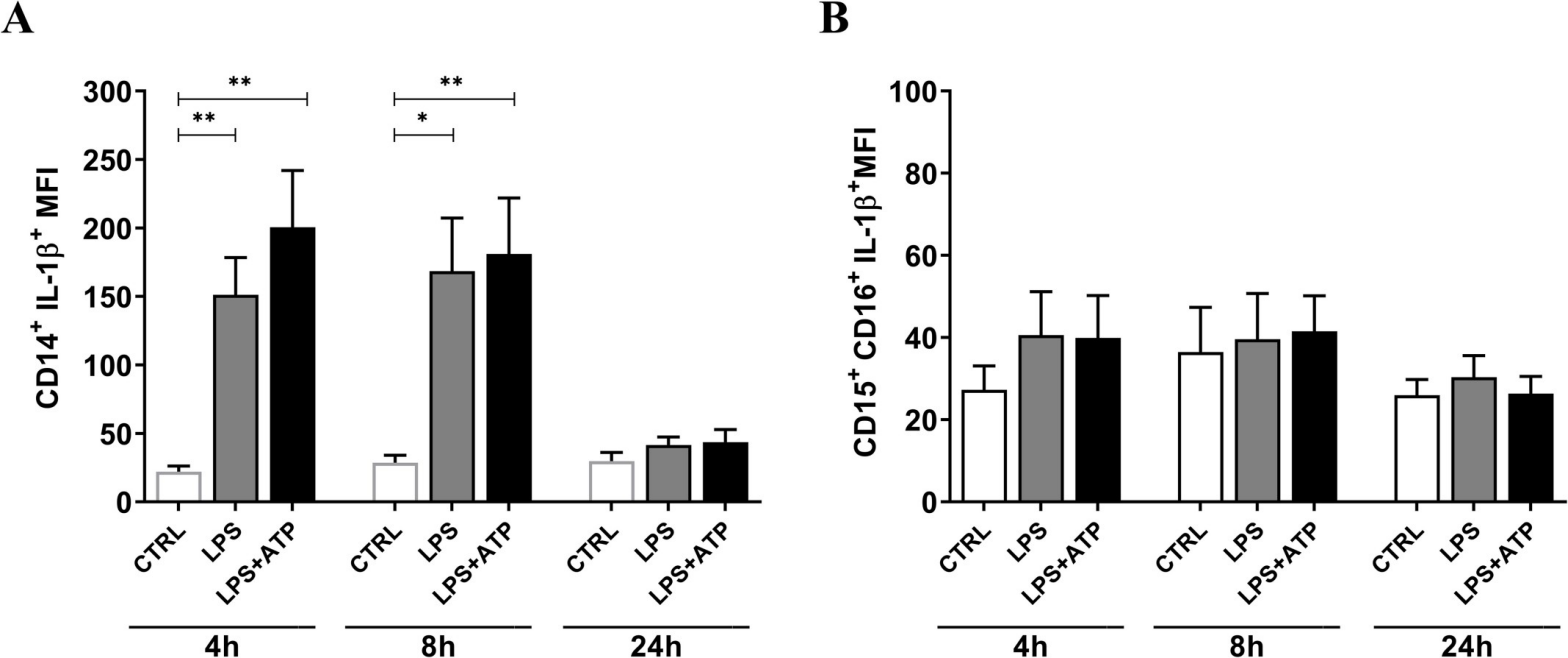
IL-1β expression in monocytes and neutrophils in whole blood. Mean fluorescence intensity (MFI) value ± SEM of IL-1β expression for each stimulation conditions (CTRL: open bars, LPS: grey bars, LPS+ATP: black bars) after 4, 8 and 24h in (A) CD14^+^ monocytes, (B) CD15^+^CD16^+^ neutrophils. n = 6. *p<0.05 and **p<0.008 analyzed by mixed effects analysis with Tukey’s multiple comparison test.

IL-1β is a potent pro-inflammatory cytokine produced by cells of the innate immune system. The IL-1β production in innate immune cells in whole blood was performed by co-staining for intracellular IL-1β and for cell surface markers used for characterization of monocytes, neutrophils and dendritic cells as explained in materials and methods. The pattern of IL-1β induction in CD14^+^ monocytes resembles the release of IL-1β in whole blood over a period of 24h, with statistically significant increase of IL-1β in LPS and LPS+ATP compared to unstimulated control at 4 and 8h (**Fig 2A**), indicating that monocytes are a major contributor to the responses in whole blood to LPS and LPS+ATP. Flow cytometry evaluation of IL-1β expression in CD15^+^CD16^+^ neutrophils showed that inflammasome activating condition (LPS+ATP) did not induce significant IL-1β compared to control or LPS stimulated whole blood samples (**Fig 2B**), while myeloid dendritic cells to a minor extend contribute to IL-1β processing in whole blood assay (**S4 Fig**).

### NLRP3 inflammasome involvement in IL-1β generation

**Fig 3:**
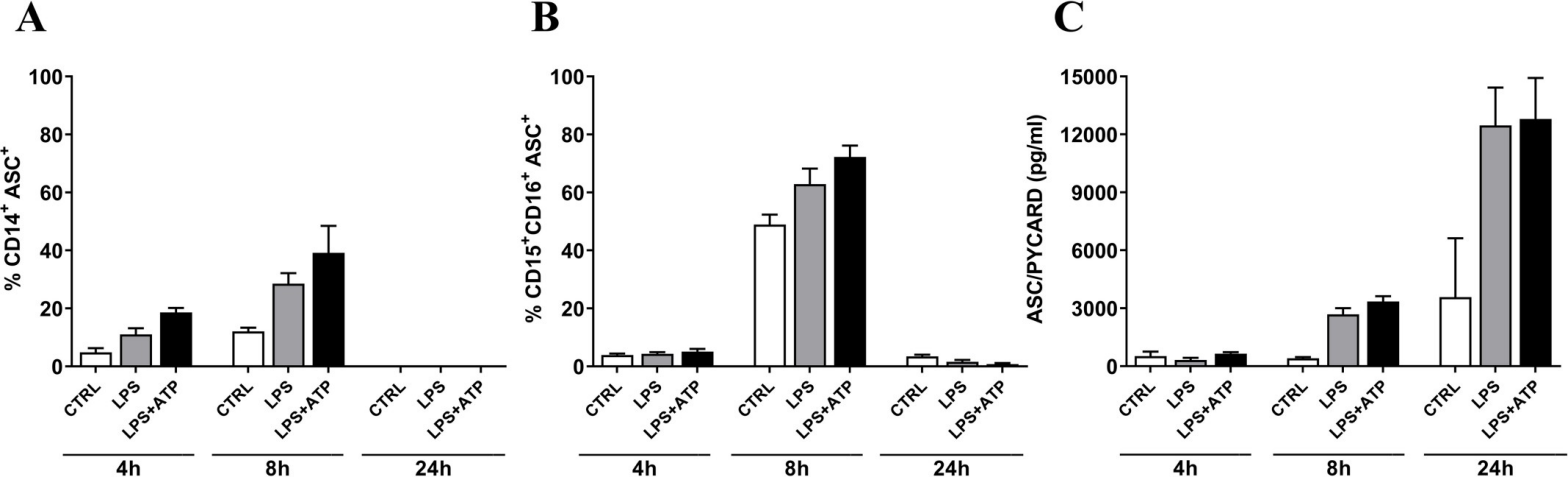
ASC detection in whole blood. Percentage ASC expression for each stimulation conditions (CTRL: open bars, LPS: grey bars, LPS+ATP: black bars) after 4, 8 and 24h in CD14^+^ monocytic population (**A**) and CD15^+^CD16^+^ neutrophil population (**B**). (**C**) ASC/PYCARD detection in plasma upon inflammasome activation with LPS or LPS+ATP. Data are expressed as mean value ± SEM of each stimulation condition, n = 2 (**A, B**) and n = 6 (**C**).

To elucidate the role of NLRP3 the cells were stained with apoptosis-associated speck-like protein containing a caspase recruitment domain (ASC) together with markers for characterization of monocytes and neutrophils. At 4h after stimulation the monocytic cell population showed slight increase of ASC expression in LPS+ATP stimulated cells compared to control with further increase at 8h (**Fig 3A**). A different profile of ASC expression was observed for the neutrophils, with high levels at 8h in both control as well as LPS or LPS+ATP stimulated samples (**Fig 3B**). And for both population at 24h there were almost none detectable levels (**Fig 3A and 3B**). Additionally, release of ASC into the supernatant was also tested with detectable levels in stimulated conditions at 8h (3000 pg/ml) and further up-regulated 4-fold at 24h (12000 pg/ml) (**Fig 3C**). To further investigate the involvement of NLRP3 the well-known inhibitor MCC950 was applied only at the 4h condition, where a significant inhibitory effect of 65% was observed for the release of IL-1β (**S5A Fig**) and 90% for the release of ASC/PYCARD (**S5B Fig**) compared to the LPS+ATP condition.

### Monocytes display phenotype shift upon inflammasome activation in whole blood cultures

**Fig 4:**
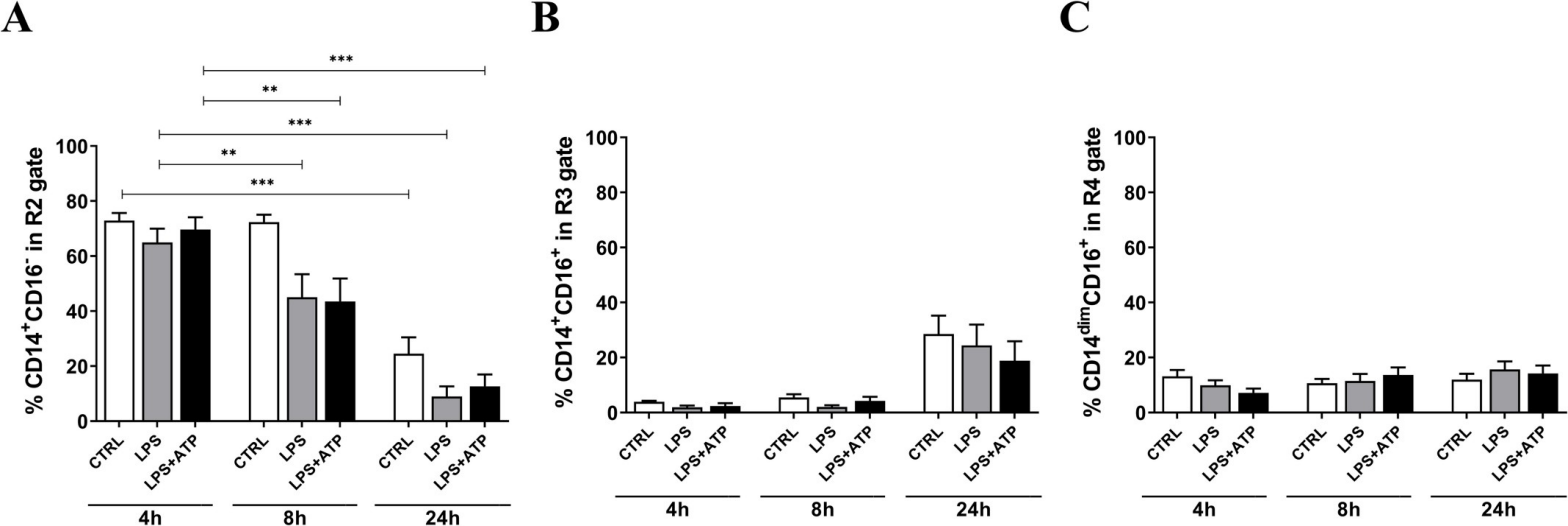
Monocytes phenotypically change during stimulation over time. Percentage expression of CD14^+^CD16^−^ monocytes (A), CD14^+^CD16^+^ monocytes (B) and CD14^dim^CD16^+^ monocytes (C). Data are expressed as mean value ± SEM of each stimulation condition, n = 6. **p<0.005 and ***p<0.0001 analyzed by mixed effects analysis with Dunnett’s multiple comparison test.

Human blood monocytes can be classified into three distinct populations, classical CD14^+^CD16^−^ monocytes, intermediate CD14^+^CD16^+^ and non-classical CD14^dim^CD16^+^ monocytes [31]. Each class contributes to the inflammatory response in different manners due to differences in expression of TLRs and cytokine/chemokine responses [32]. Flow cytometry analysis in basal, not manipulated whole blood confirmed the existence of all three monocytic subsets with distribution similar to previous reports (**S6 Fig**) [33]. Treatment with LPS or LPS+ATP did not induce prominent changes in CD14 and CD16 expression in all three subsets after 4h compared to control (**Fig 4**). At 8h the only change observed was of the CD14^+^CD16^−^ cell phenotype significantly decreased in both stimulated conditions (LPS and LPS+ATP) but not in the control (**Fig 4A**). And two subsets of the monocytes changed after 24h, namely the number of CD14^+^CD16^−^ cells (**Fig 4A**) declined, which was accompanied by the increase of CD14^+^CD16^+^ cells (**Fig 4B**) at the same time-point (see also **S7 Fig**). The number of CD14^dim^CD16^+^ cells was unaltered during the 24h experimental period. Intracellular staining for IL-1β in two donors demonstrated that CD14^+^CD16^−^ are predominant source of IL-1β within the monocytic subsets (**S8 Fig**).

### IL-1β and IL-18 release is inhibited by caspase inhibitors

**Fig 5:**
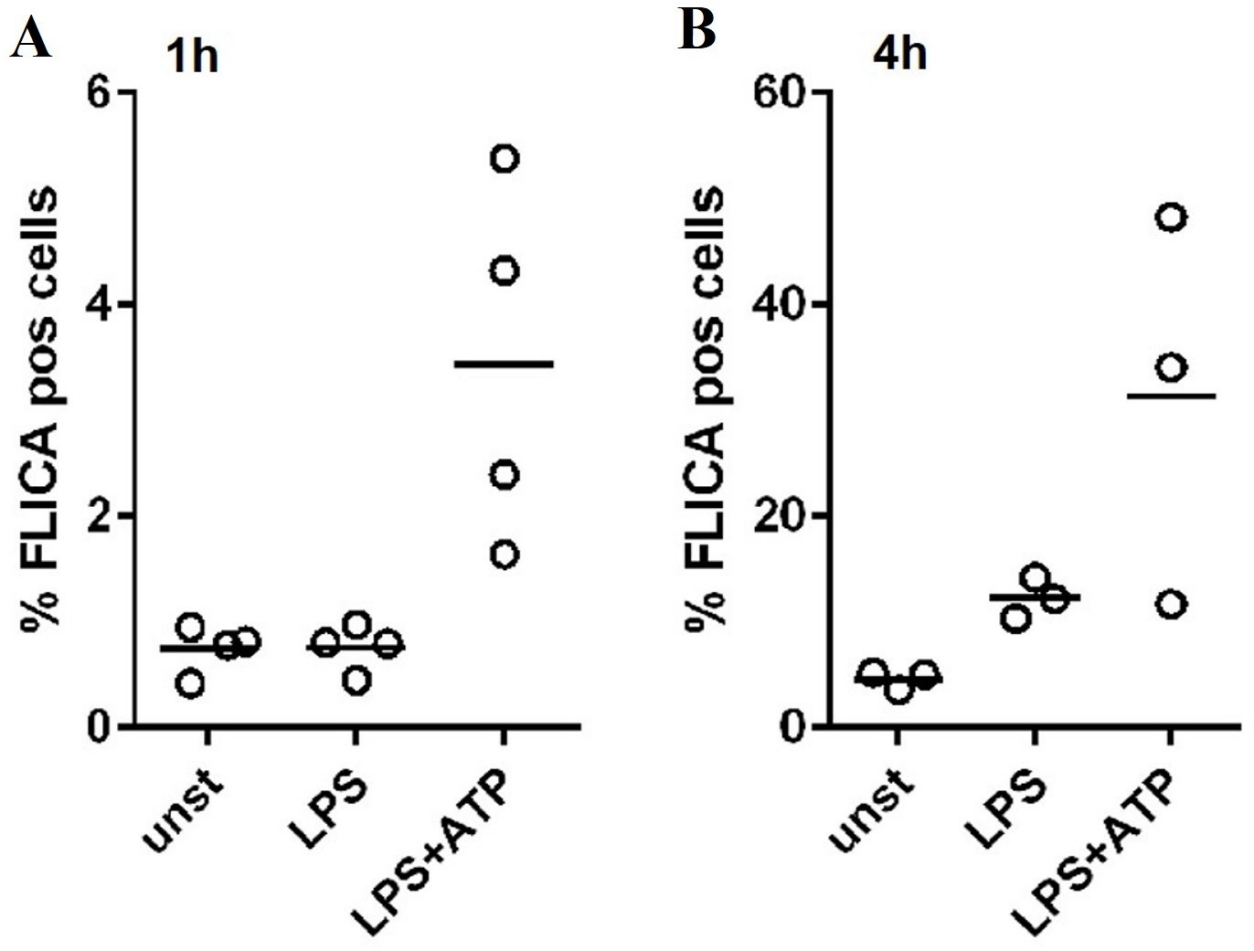
Caspase activation in whole blood assay. FAM-YVAD-FMK (FLICA) binding to active caspase-1 in monocytes after 1h (A) and 3h stimulation (B). Data are expressed as % of FLICA^+^ cells in whole blood, n = 4 (A) and n = 3 (B).

Upon confirmation that monocytes produce IL-1β, caspase-1/4/5 activity was investigated in this cell population using FAM-YVAD-FMK, a fluorescent probe that binds active caspase-1/4/5, referred to here as FLICA. CD14^+^ monocytes were analyzed by flow cytometry after whole blood stimulation with LPS or LPS+ATP. After stimulation for 4h, LPS induced 12% ± 2% FLICA^+^ monocytes, while LPS+ATP treatment induced 31% ± 15% FLICA^+^ monocytes (**Fig 5B**). In a separate experiment, stimulation for 1h showed only FLICA positive monocytes after LPS+ATP exposure (**Fig 5A**). These data demonstrate clearly caspase activation by monocytes in whole blood cultures and indicates that caspase-1 is not constitutively active in monocytes.

**Fig 6:**
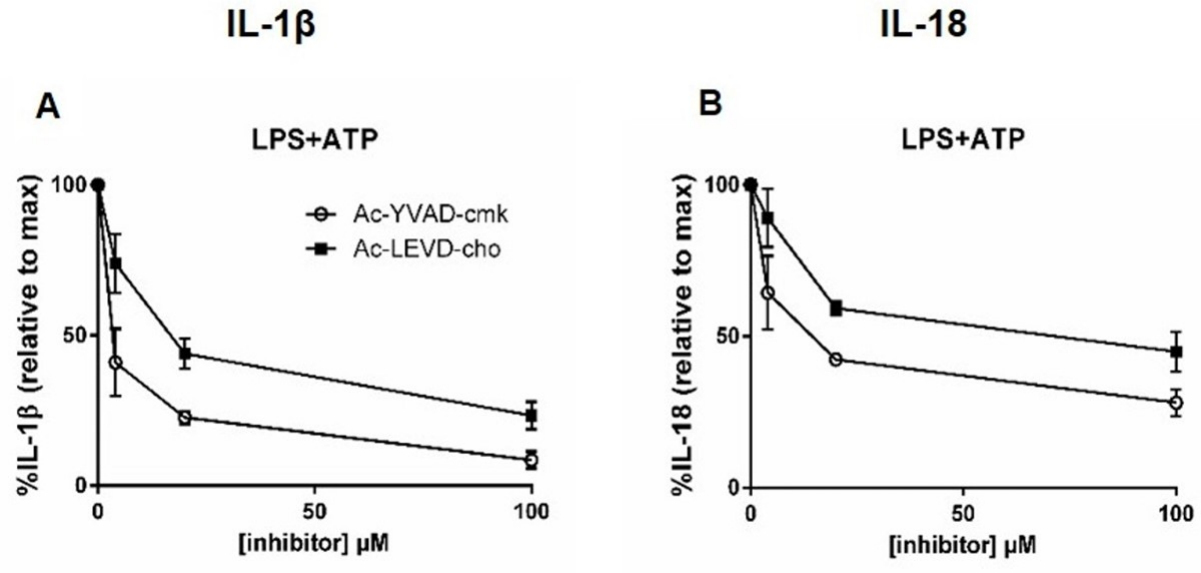
The effect of caspase-1 and caspase-4 inhibition on IL-1β and IL-18 secretion. Caspase inhibition of IL-1β release upon LPS+ATP (A) stimulation. Caspase inhibition of IL-18 release upon LPS+ATP stimulation (B). Data are expressed as mean ± % CV of duplicates from three independent experiments.

To confirm that in our whole blood cultures the intracellular IL-1β cleavage occurs as result of caspase activation after inflammasome assembly, we performed a whole blood experiment with caspase inhibitors Ac-YVAD-cmk and Ac-LEVD-cho. Whole blood was incubated for 1h with 4, 20 or 100 μM Ac-YVAD-cmk (preferential caspase-1 recognition site) or Ac-LEVD-cho (preferential caspase-4 recognition site) prior to inflammasome stimulation [34]. Following incubation with the caspase inhibitors, cultures were stimulated with LPS+ATP in order to specifically activate caspase-1 [35, 36]. Both inhibitors inhibited IL-1β release after LPS+ATP stimulation (**Fig 6A**) and IL-18 release after LPS+ATP stimulation (**Fig 6B**), showing caspases-1 and 4/5 were activated after stimulation in whole blood. These results were consistent in five additional blood cultures from healthy donors (**S9A, S9B and S9C Fig**). The fact that both inhibitors inhibited IL-1β and IL-18 release after inflammasome stimulation indicates that both caspase-1 and caspase-4 play a role in the processing of these cytokines. The effect of both inhibitors on TNFα and IL-6 release after LPS and LPS+ATP was measured as a control (**S9D Fig**), and here no effect of the inhibitors was observed.

## Discussion

Altered inflammasome activity has been implicated in multiple diseases including pseudogout, asbestosis, Alzheimer’s, cancer, atherosclerosis, and type 2 diabetes mellitus [19, 27, 37]. In order to set up an easy and robust assay for inflammasome research, we explored the use of *ex vivo* whole blood cultures as a tool to study inflammasome activation resembling the *in vivo* circumstances as much as possible. The isolation of PBMCs or other cells, such as macrophages from peripheral blood, may induce unwanted side effects such as a higher level of apoptosis in isolated PBMCs compared to whole blood [38]. Moreover, in whole blood cultures the influence of sample handling-related cell activation is minimal, so this is the most natural milieu to study cytokine production *in vitro* [39].

First, we optimized our conditions by evaluating the optimal time-frame for inflammasome activation. After priming human whole blood with LPS for periods up to 24h, subsequent co-stimulation with ATP did not result in increased secretion of IL-1β and IL-18 as compared to shorter LPS incubations (4 and 8h). Evaluation of the secretion of potential inhibitory mechanisms such as IL-1sRII and IL-18BPa did not demonstrate any related kinetic patterns associated with limited time-frame of increased IL-1β production, suggesting these are not critical factors in the down-regulation of IL-1β. To further investigate what underlies IL-1β and IL-18 decrease at 24h, we studied the effect of LPS priming and ATP pulse application on viability of monocytes and neutrophils. Although the number of CD14^+^ monocytes declined for certain conditions, yet the viability of cells is not significantly affected as measured by staining for annexin V/PI. Similar results were obtained for CD15^+^CD16^+^ neutrophils indicating that cell death is not a reason for reduction of IL-1β. Preserved cell viability in our experimental setting is based on use of low LPS and ATP concentrations (2 ng/mL LPS + 5 mM ATP) as compared to studies that routinely have used higher concentrations (1–2 μg/mL LPS + 1–5 mM ATP) [40, 41]. This is in accordance with previously reported cell viability in similar settings [40, 42, 43]. Taken together, priming cells with LPS for 24h and incubating with ATP for 30 min results in diminished IL-1β and IL-18 induction indicating that a negative feedback mechanism is mainly responsible for the observed time-response courses in our model. This is also in line with the growing paradigm for negative feedback initiated by LPS signaling patterns in general [44–46]. In addition, a recent study of Gurung et al. has identified IL-10 as a soluble secreted factor that acts as a negative feedback loop to dampen NLRP3 inflammasome activation [47].

It is well acknowledged that monocytes are major source of IL-1β in circulation although recently it has been shown that neutrophils also contribute to the total pool of circulating IL-1β [48]. Our data indeed suggests that the majority of IL-1β is produced by monocytes and neutrophils. Detectable intracellular MFI levels of IL-1β in neutrophils irrespective of stimulation suggests that even a minimal manipulation of whole blood might trigger neutrophil activation, followed by increase of intracellular IL-1β levels or that neutrophils are constitutively loaded with IL-1β. Inflammasome activation in whole blood was confirmed by FLICA staining but evaluation of the level of inflammasome activation in monocytes vs. neutrophils remains to be investigated. With direct cellular staining of ASC and quantification of ASC levels in supernatant we were able to detect levels of speck formation and thereby verify the involvement of NLRP3 in the generation of IL-1β. Consistent with the findings by Stehlik et al. [49] we also show that ASC enhances IL-1β secretion in culture supernatant at low concentration observed for the monocytes at 4 and 8h while having a suppressing function at high concentration shown at 24h secreted in the supernatant.

Inflammatory ligands to TLRs often induce signaling events that result in changes of the expression of cell-characteristic surface markers and induction of different phenotype in blood cells and monocytes are not an exemption of this pattern [33, 50]. It is acknowledged that the monocytes are composed of three cell subsets based on the expression of CD14 and CD16: classical CD14^+^CD16^−^, intermediate CD14^+^CD16^+^, and non-classical CD14^dim^CD16^+^ monocytes [33]. Each of these subsets possesses distinct biological functions and different responses to inflammation [31, 32]. Flow cytometry assessment of monocytic populations presented in our study confirmed the existence and distribution of all three monocytic subsets are in line with previously reports [51]. Short (4h) incubations do not appear to induce major loss or gain of expression of monocyte markers CD14 and CD16, irrespectively of experimental condition. Similar observation with LPS stimulation for 4h was reported by others [52]. However, prolonged exposure to experimental conditions up to 24h induced a drop in the number of classical CD14^+^CD16^−^ monocytes and an increase of intermediate CD14^+^CD16^+^ cells. This re-distribution of the monocytic subsets suggest that the classical monocytes shift their phenotype towards the intermediate phenotype, because it is rather unlikely that the reduction in the number of classical and the increase of intermediate monocytes are events related to the cell death of the former and proliferation of the latter subset. The reduction of IL-1β at 24h coincidences with the phenotypical shift observed at the same time-point implying that this cytokine might be involved in the appearance of higher number of intermediate CD16^+^ monocytes, since it has been reported that IL-1β induces CD16 expression in macrophages [53]. This intermediate subset is acquiring a more phagocytic phenotype due to the expression of CD16 [54] and has a different cytokine profile compared to classical and non-classical monocytes [31, 55]. Differences between monocytic cells were also observed with respect to the synthesis of IL-1β in this study. The pattern of IL-1β intracellular staining was very similar in tested donors and revealed that the classical monocytes are the predominant subset that synthesize most of IL-1β. Intermediate monocytes also contribute to the total IL-1β while the non-classical monocytes produce less IL-1β compared to the other monocyte subsets. Consistent with previous reports, we observed that the IL-1β production by monocyte subsets is LPS induced and augmented by ATP [56, 57].

To show that caspases are activated and necessary for IL-1β and IL-18 cleavage in our whole blood model, we performed experiments using Ac-YVAD-cmk and Ac-LEVD-cho. Unfortunately no specific caspase inhibitors are available, Ac-YVAD-cmk preferentially inhibits caspase-1 but also inhibits caspases 4 and 5 [58], whereas Ac-LEVD-cho preferentially inhibits caspase-4, but also caspases 1 and 5 [34]. Both inhibitors inhibited IL-1β after LPS stimulation to a similar extent, however Ac-YVAD-cmk inhibits IL-1β and IL-18 release more strongly after LPS+ATP stimulation (**Fig 6**). Another interesting finding is that IL-1β is released after stimulation with LPS alone (**Fig 1**), whereas IL-18 needs a secondary ATP trigger to be released (**S2 Fig**). Recently it was shown that LPS activates the non-canonical pathway acting via caspase-4/5 without the need of a secondary trigger [59]. Vigano et al. show that the internalization of LPS via CD14/TLR4 activates a one-step inflammasome activation via syk and a Ca^2+^ flux, that results in the processing and release of IL-1β via caspase-4/5 [42]. The involvement of the non-canonical inflammasome pathway explains both why IL-1β is released after LPS stimulation as well as why Ac-LEVD-cho is a less potent IL-1β and IL-18 inhibitor after LPS+ATP stimulation.

Several studies have shown benefits of stimulation of whole blood culture over purified cells for *in vitro* analysis of immune cell function including inflammasome response [60, 61].

In the present study, we show that the *ex vivo* whole blood stimulation is a simple and robust model for inflammasome activation, with detection of NLRP3 inflammasome signaling, caspase activation followed by IL-1β expression and secretion in human whole blood with minimal manipulation. This assay enables timely identification of possible overt inflammasome activation by novel therapeutics in human subjects prior application in first-in-human studies. Furthermore, this test by mimicking the physiological milieu to great extend is a reliable platform to study novel drugs that aim to suppress the inflammasome activation in pathological conditions characterized by profound production of IL-1β.

## Supporting information

S1 Fig Gating strategies for flow cytometry analysis. **(Panel A)** Gating strategy for assessment of IL-1β expression in monocytes and neutrophils, example from donor 1 CTRL 4h. (**A**) Via side scatter-area (SSC-A) and CD45 expression monocytes and granulocytes were gated. (**B**) Expression of CD15 and CD16 were used to identity neutrophils in the granulocyte gate. (**C**) Mean fluorescence intensity of IL-1β expression in CD15^+^CD16^+^ neutrophils. (**D**) CD14 was used to identify classical monocytes in the monocyte gate. (**E**) Mean fluorescence intensity of IL-1β expression in CD14^+^ monocytes. **(Panel B)** Gating strategy for monocyte phenotype determination, example from donor 1. (**A)** Via side scatter-area (SSC-A) and CD45 expression monocytes was gated. (**B)** CD14 and CD16 were used to identify different sub-populations of monocytes as classical (CD14^+^CD16^−^), intermediate (CD14^+^CD16^+^) and non-classical (CD14^−^CD16^+^). **(Panel C)** Gating strategy for assessment of IL-1β expression in monocytes and dendritic cells, monocytes and mDCs and pDCs are indentified as being HLA-DR^+^ and lineage negative. Monocytes are gated as CD14^+^, while mDCs and pDCs are CD14^−^. Subsequently, pDCs are identified as being CD123^+^, CD11c^−^, while mDCs are CD123^−^ and CD11c^+^. Data analysis was performed using Flowjo version 10 software.
(TIF)

S2 Fig Dynamics and kinetics of pro-inflammatory mediators after inflammasome activation with LPS or LPS+ATP. IL-18, IL-6, IL-8 and TNFα levels in supernatant collected after LPS and LPS+ATP treatment of whole blood cultures (n = 1). Data are expressed as mean ± % CV of duplicate conditions of one experiment.
(TIF)

S3 Fig Dynamics and kinetics of IL-18BPa and IL-1sRII after inflammasome activation. Assessment of IL-18BPa and IL-1sRII, inhibitors for IL-18 and IL-1β, respectively, in supernatant after LPS and LPS+ATP treatment in whole blood cultures (n = 1). Data are expressed as mean ± % CV of duplicate conditions of one experiment.
(TIF)

S4 Fig IL-1β production by monocytes and myeloid dendritic cells (mDC). Data are expressed as % of IL-1β positive cells as determined by expression of monocytic and dendritic cells markers using flow cytometry (n = 3).
(TIF)

S5 Fig Inhibition of NLRP3 effectively suppress IL-1β secretion. (**A**) Inhibitory effect of IL-1β secretion and (**B**) of ASC/PYCARD secretion. Data are expressed as mean value ± SEM, n = 3. *p<0.05 analyzed by paired two-tailed t-test.
(TIF)

S6 Fig Baseline values for monocyte subpopulations. Distribution of monocytic subsets prior stimulation (CTRL 0h). Data are expressed as mean ± SEM from six donors (n = 6).
(TIF)

S7 Fig Dynamics and kinetics of distribution of monocytic subsets during inflammasome activation. Representative dot plots of three distinctive monocyte subsets (upper left gate: CD14^+^CD16^−^, upper right gate CD14^+^CD16^+^, and lower right gate: CD14^dim^CD16^+^) enumerated after 4, 8 and 24h of stimulation with LPS or LPS+ATP by flow cytometry. CTRL 0h depicts the distribution of monocyte subsets prior stimulation and incubation.
(TIF)

S8 Fig IL-1β expression in monocyte subsets. Intracellular IL-1β expression within monocyte subsets was assessed in two donors by flow cytometry. Data are expressed as % of IL-1β positive cells of total monocytic fraction of whole blood.
(TIF)

S9 Fig Effect of caspase 1/4 inhibitors on cytokine production. Caspase inhibition of IL-1β release upon LPS (**A**) and LPS+ATP (**B**) stimulation. Caspase inhibition of IL-18 release upon LPS+ATP stimulation (**C**). Levels of IL-6 and TNFα in supernatant (**D**). Data are expressed as mean ± % CV of duplicates of 5 independent experiments.
(TIF)

## Abbreviations

ATP: adenosine 5’-triphosphate
ASC: apoptosis-associated speck-like protein containing a caspase recruitment domain
FLICA: Fluorescent Labelled Inhibitor of Caspases
IFNγ: interferon-gamma
IL: interleukin
IL-1sRII: IL-1 receptor II
LPS: lipopolysaccharide
MFI: mean fluorescence intensity
NF-κ: nuclear factor-κ
NLRP3: nucleotide binding and oligomerization domain and leucine rich-repeat-containing pyrin domain containing 3
PBMC: peripheral blood mononuclear cell
SEM: standard error of the mean
TLR: Toll-like receptor
TNFα: tumor necrosis factor alpha
