# Supplementary figures and images for: Whole blood assay as a model for in vitro evaluation of inflammasome activation and subsequent caspase-mediated interleukin-1 beta release
Source: PLoS One. 2019 Apr 8;14(4):e0214999. doi: 10.1371/journal.pone.0214999 (PMC6453527; doi:10.1371/journal.pone.0214999)

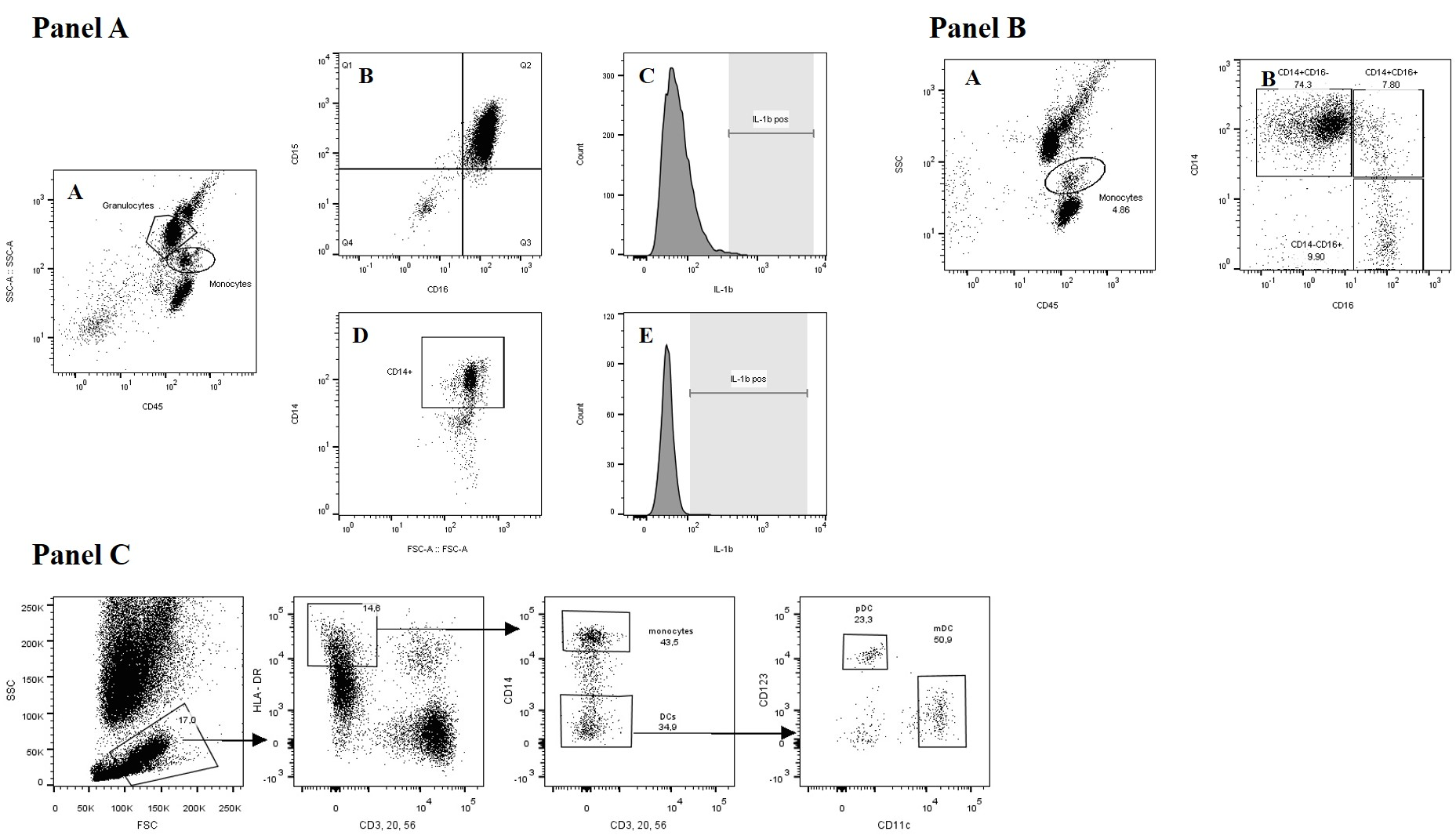

Supplement: S1 Fig — (Panel A) Gating strategy for assessment of IL-1β expression in monocytes and neutrophils, example from donor 1 CTRL 4h. (A) Via side scatter-area (SSC-A) and CD45 expression monocytes and granulocytes were gated. (B) Expression of CD15 and CD16 were used to identity neutrophils in the granulocyte gate. (C) Mean fluorescence intensity of IL-1β expression in CD15+CD16+ neutrophils. (D) CD14 was used to identify classical monocytes in the monocyte gate. (E) Mean fluorescence intensity of IL-1β expression in CD14+ monocytes. (Panel B) Gating strategy for monocyte phenotype determination, example from donor 1. (A) Via side scatter-area (SSC-A) and CD45 expression monocytes was gated. (B) CD14 and CD16 were used to identify different sub-populations of monocytes as classical (CD14+CD16−), intermediate (CD14+CD16+) and non-classical (CD14−CD16+). (Panel C) Gating strategy for assessment of IL-1β expression in monocytes and dendritic cells, monocytes and mDCs and pDCs are indentified as being HLA-DR+ and lineage negative. Monocytes are gated as CD14+, while mDCs and pDCs are CD14−. Subsequently, pDCs are identified as being CD123+, CD11c−, while mDCs are CD123− and CD11c+. Data analysis was performed using Flowjo version 10 software. (TIF) [file pone.0214999.s001.tif]

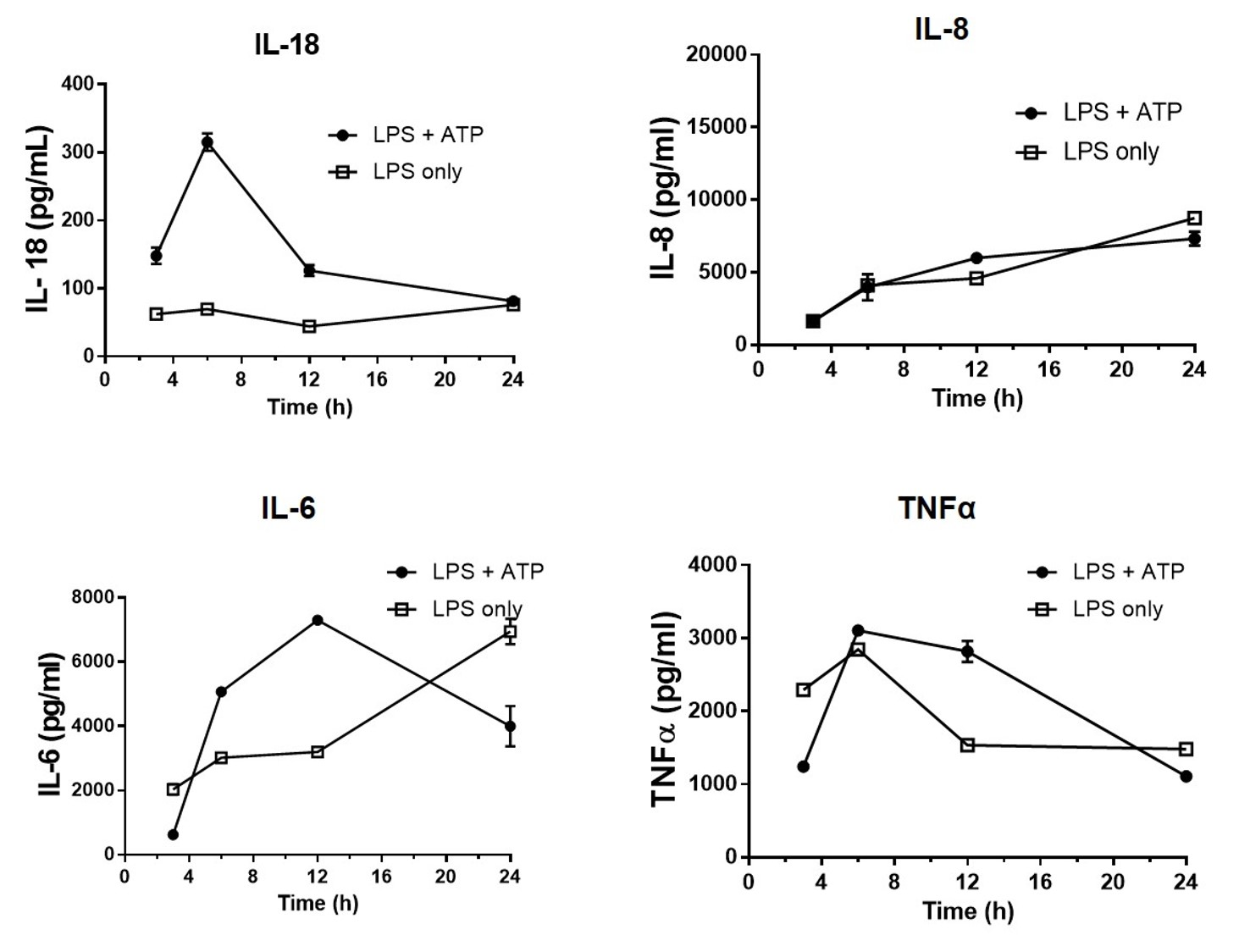

Supplement: S2 Fig — IL-18, IL-6, IL-8 and TNFα levels in supernatant collected after LPS and LPS+ATP treatment of whole blood cultures (n = 1). Data are expressed as mean ± % CV of duplicate conditions of one experiment. (TIF) [file pone.0214999.s002.tif]

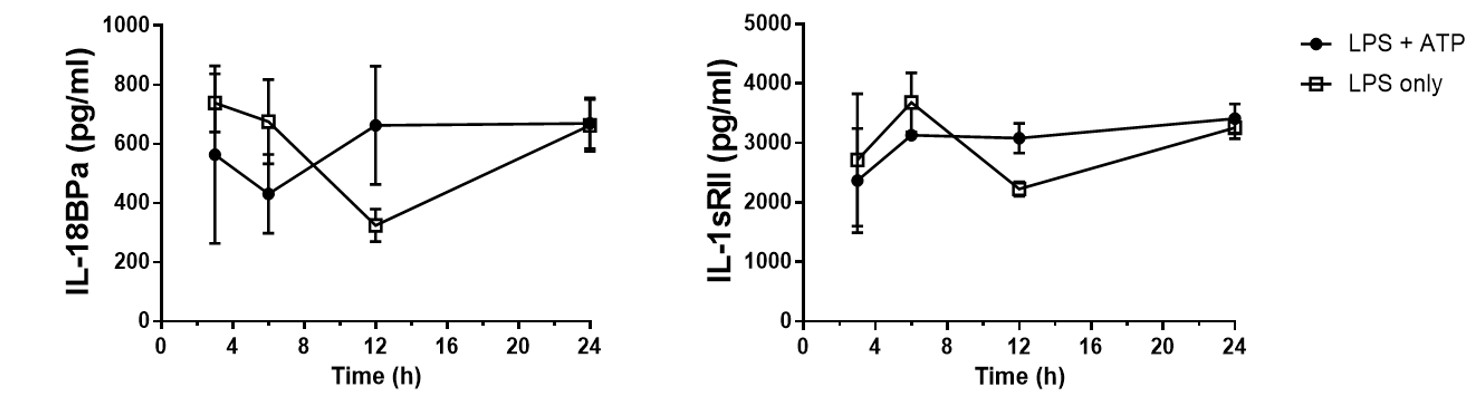

Supplement: S3 Fig — Assessment of IL-18BPa and IL-1sRII, inhibitors for IL-18 and IL-1β, respectively, in supernatant after LPS and LPS+ATP treatment in whole blood cultures (n = 1). Data are expressed as mean ± % CV of duplicate conditions of one experiment. (TIF) [file pone.0214999.s003.tif]

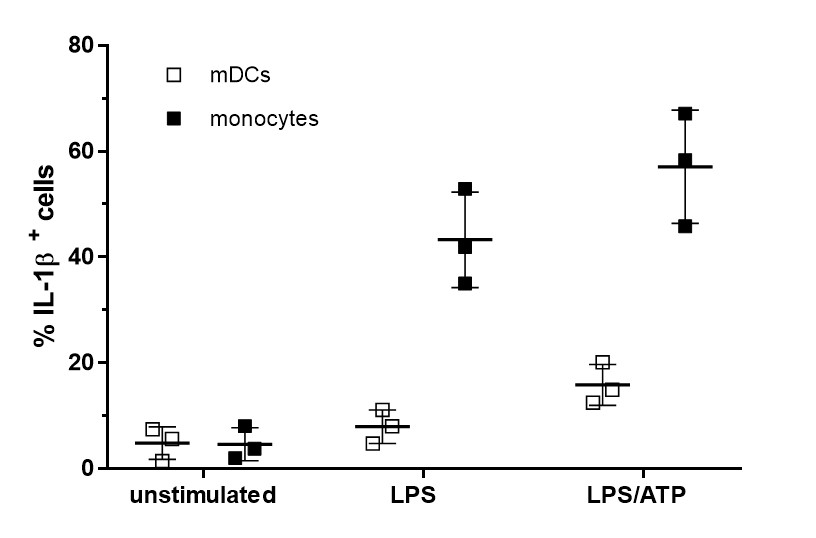

Supplement: S4 Fig — Data are expressed as % of IL-1β positive cells as determined by expression of monocytic and dendritic cells markers using flow cytometry (n = 3). (TIF) [file pone.0214999.s004.tif]

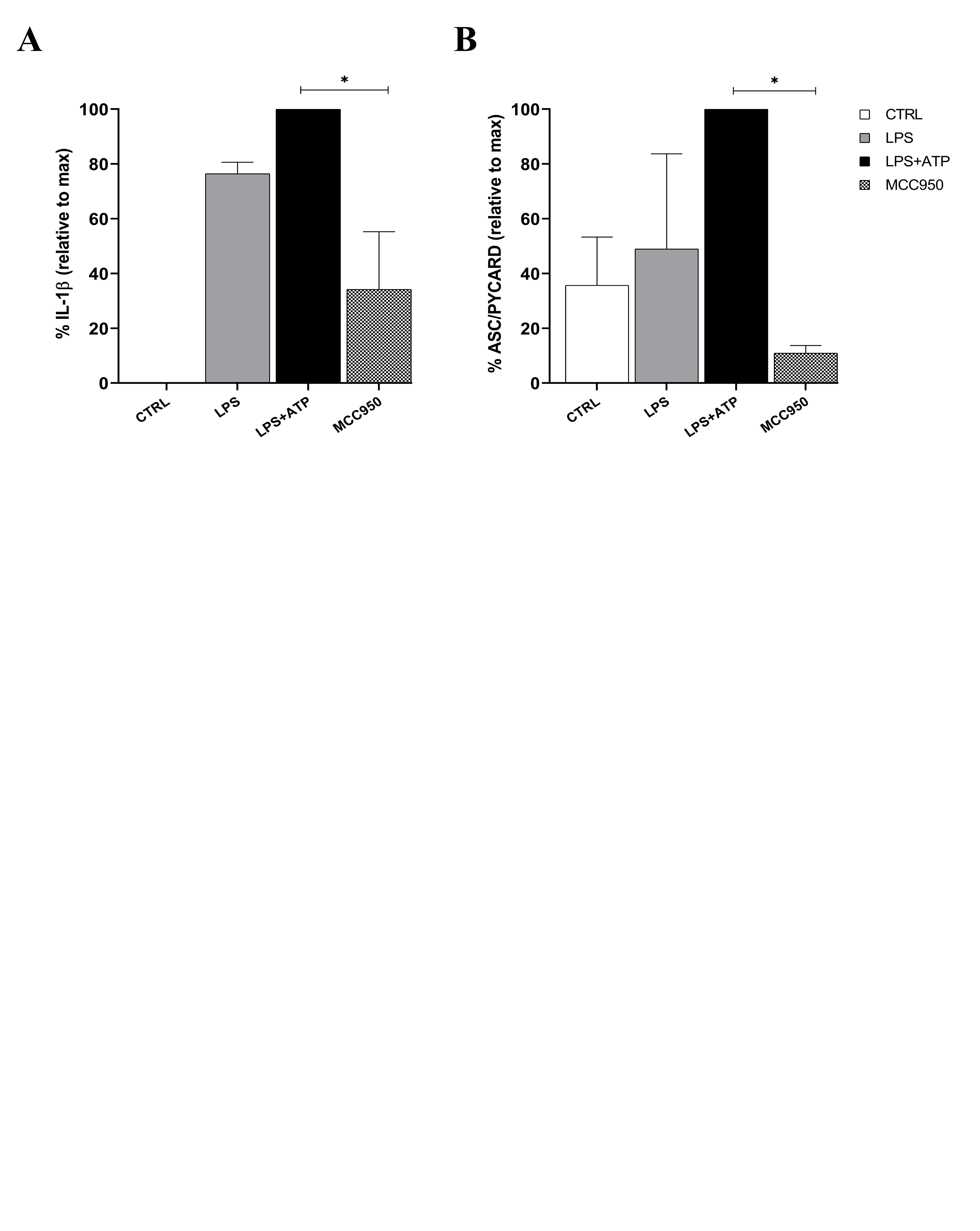

Supplement: S5 Fig — (A) Inhibitory effect of IL-1β secretion and (B) of ASC/PYCARD secretion. Data are expressed as mean value ± SEM, n = 3. *p<0.05 analyzed by paired two-tailed t-test. (TIF) [file pone.0214999.s005.tif]

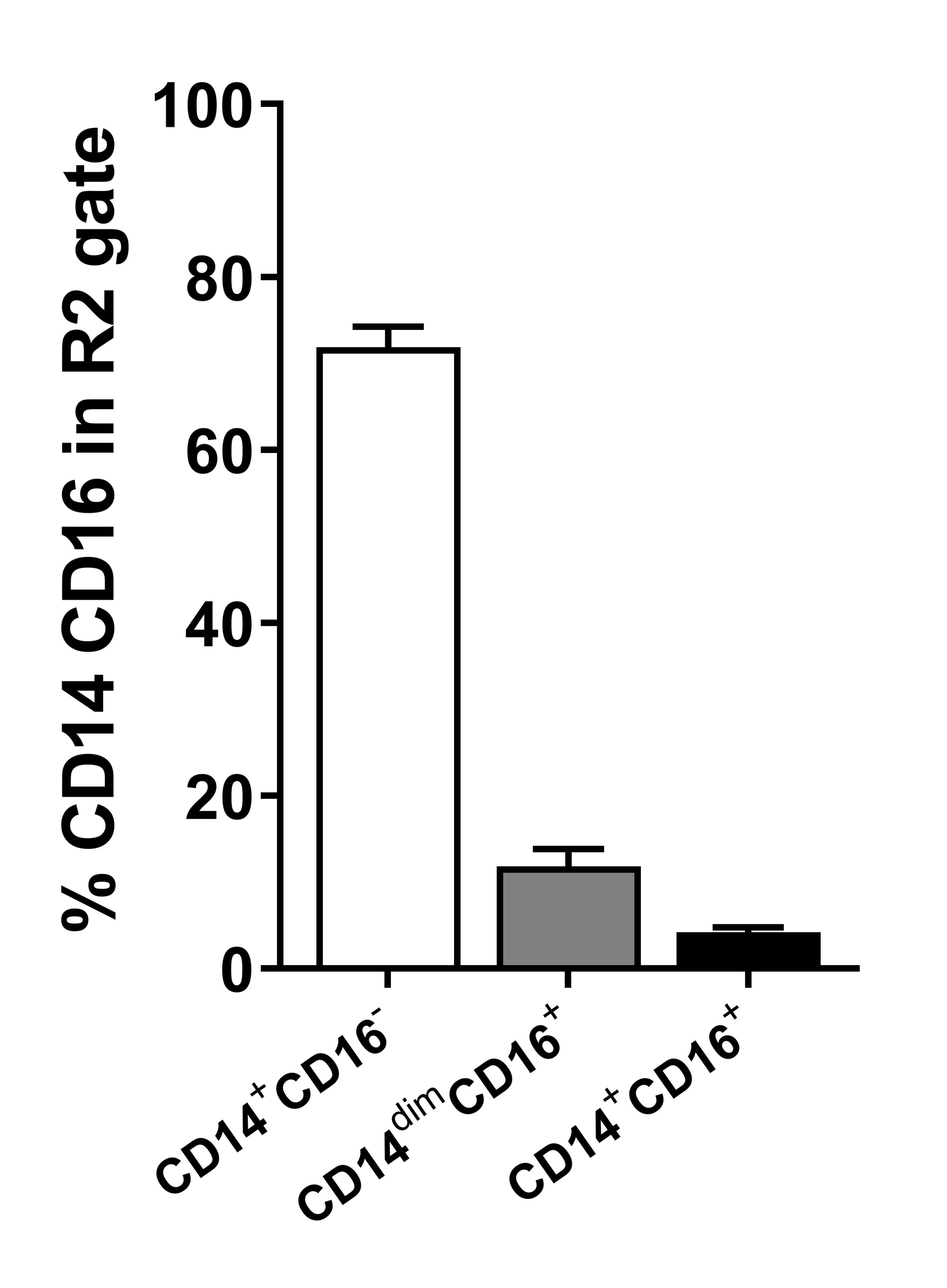

Supplement: S6 Fig — Distribution of monocytic subsets prior stimulation (CTRL 0h). Data are expressed as mean ± SEM from six donors (n = 6). (TIF) [file pone.0214999.s006.tif]

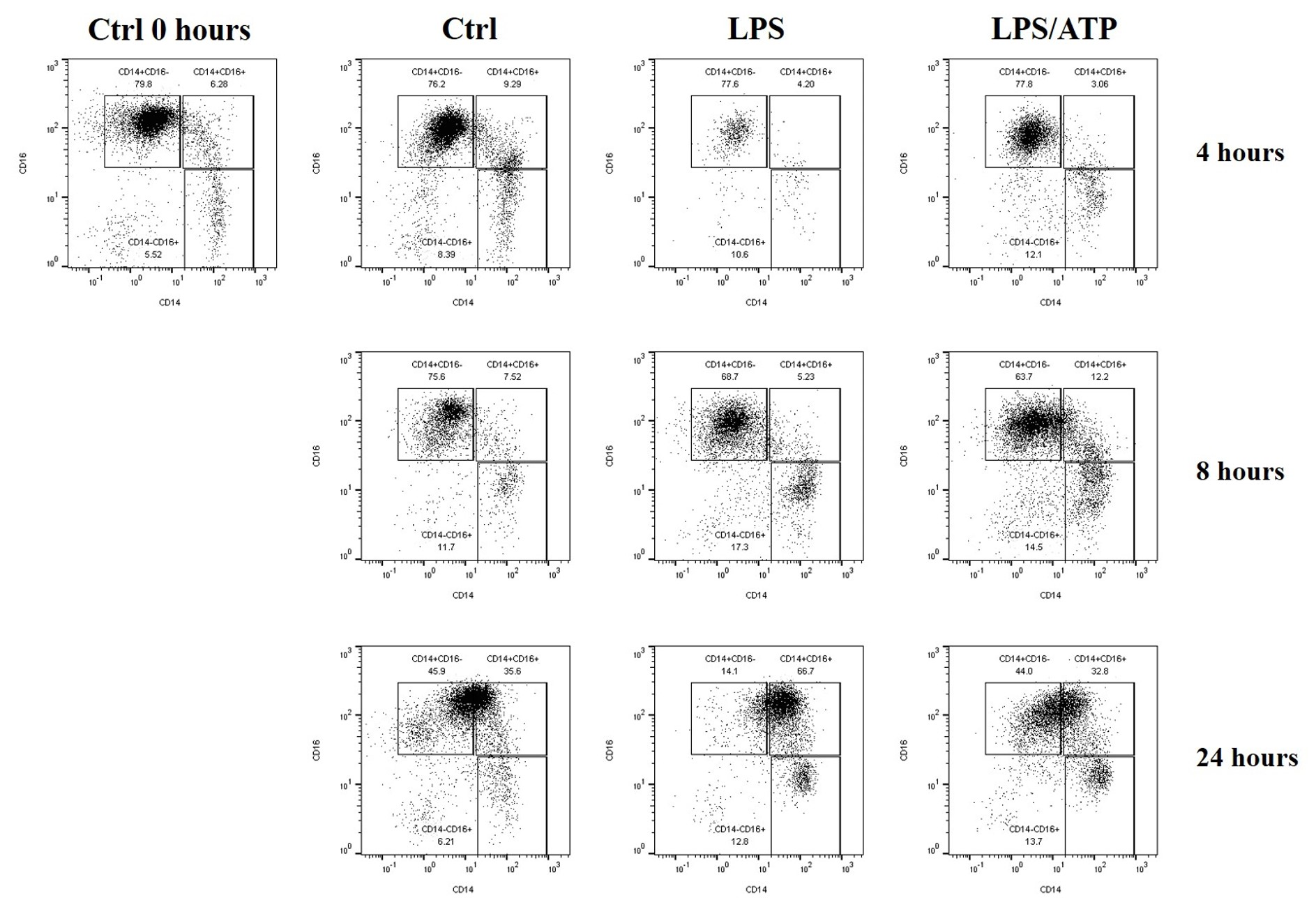

Supplement: S7 Fig — Representative dot plots of three distinctive monocyte subsets (upper left gate: CD14+CD16−, upper right gate CD14+CD16+, and lower right gate: CD14dimCD16+) enumerated after 4, 8 and 24h of stimulation with LPS or LPS+ATP by flow cytometry. CTRL 0h depicts the distribution of monocyte subsets prior stimulation and incubation. (TIF) [file pone.0214999.s007.tif]

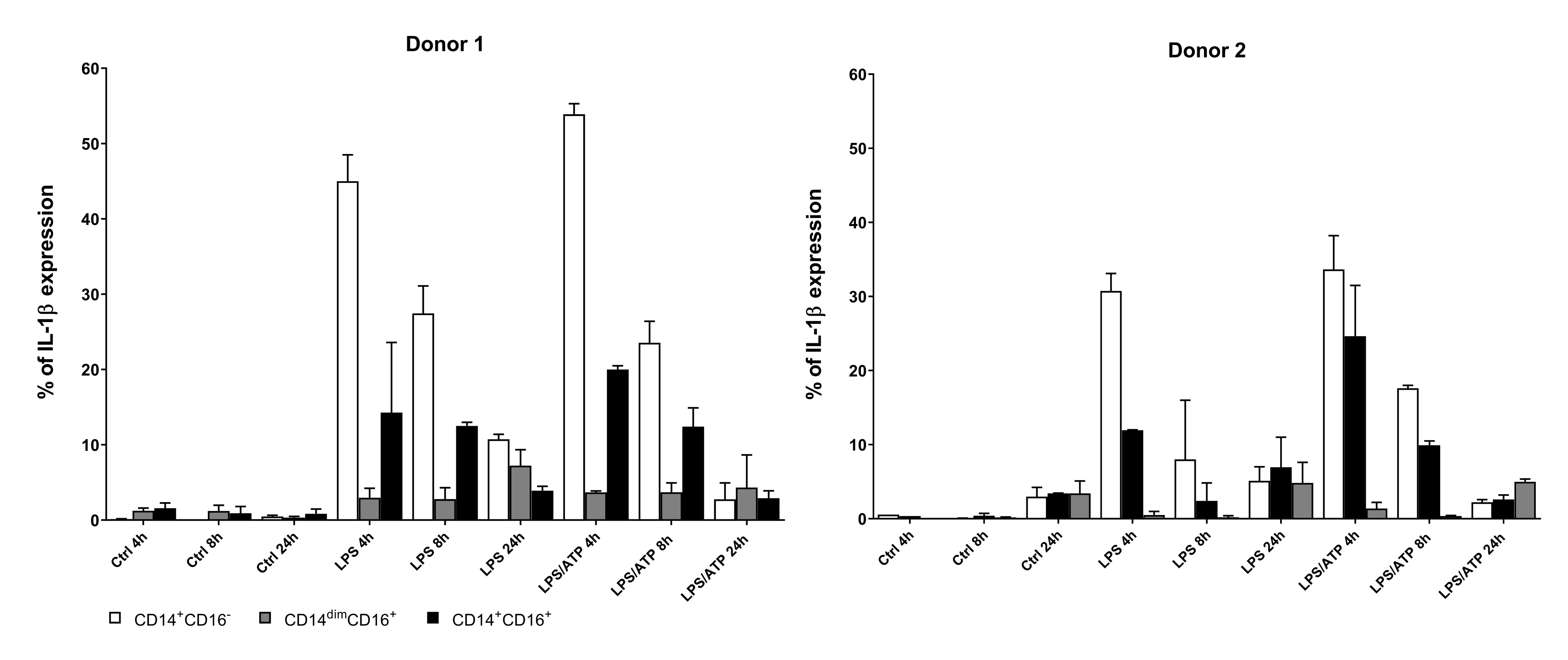

Supplement: S8 Fig — Intracellular IL-1β expression within monocyte subsets was assessed in two donors by flow cytometry. Data are expressed as % of IL-1β positive cells of total monocytic fraction of whole blood. (TIF) [file pone.0214999.s008.tif]

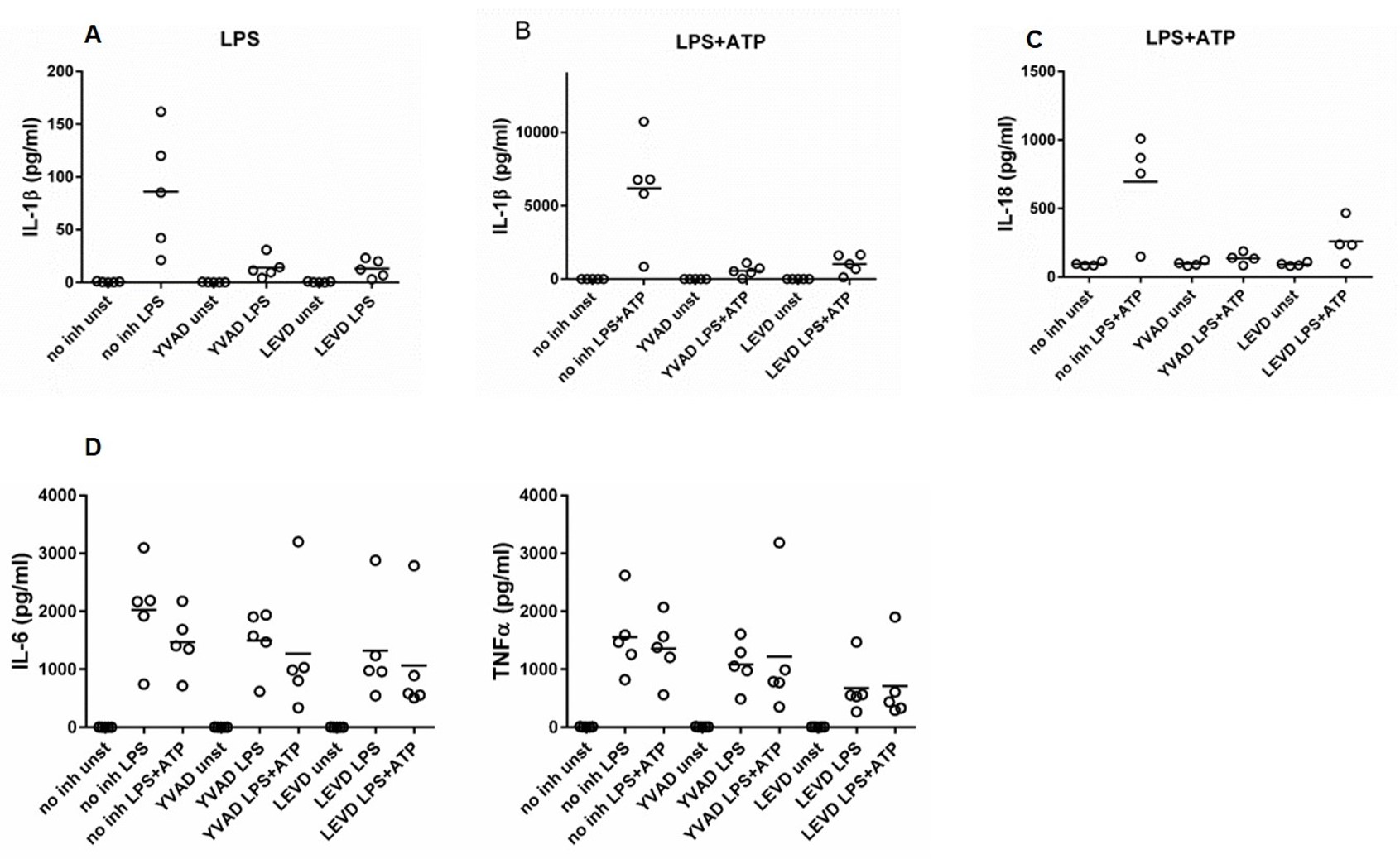

Supplement: S9 Fig — Caspase inhibition of IL-1β release upon LPS (A) and LPS+ATP (B) stimulation. Caspase inhibition of IL-18 release upon LPS+ATP stimulation (C). Levels of IL-6 and TNFα in supernatant (D). Data are expressed as mean ± % CV of duplicates of 5 independent experiments. (TIF) [file pone.0214999.s009.tif]
